# Psychiatric Acute Day Hospital as an Alternative to Inpatient Treatment

**DOI:** 10.3389/fpsyt.2020.00471

**Published:** 2020-05-25

**Authors:** Karsten Heekeren, Sofia Antoniadis, Benedikt Habermeyer, Caitriona Obermann, Matthias Kirschner, Erich Seifritz, Wulf Rössler, Wolfram Kawohl

**Affiliations:** ^1^Department of Psychiatry, Psychotherapy and Psychosomatics, University of Zurich, Zurich, Switzerland; ^2^Department of Psychiatry and Psychotherapy I, LVR-Hospital Cologne, Cologne, Germany; ^3^Department of Psychiatry and Psychotherapy, Psychiatric Services Aargau, Brugg, Switzerland; ^4^McConnell Brain Imaging Centre, Montréal Neurological Institute, McGill University, Montreal, QC, Canada; ^5^Department of Psychiatry and Psychotherapy, Charité University Medicine, Berlin, Germany

**Keywords:** psychiatric day hospital, inpatient, RCT, treatment outcome, treatment costs

## Abstract

For the first time in the Swiss health care system, this evaluation study examined whether patients with acute psychiatric illness who were admitted for inpatient treatment could be treated in an acute day hospital instead. The acute day hospital is characterized by the possibility of direct admission of patients without preliminary consultation or waiting time and is open every day of the week. In addition, it was examined whether and to what extent there are cost advantages for day hospital treatment. Patients who were admitted to the hospital with a referral to an inpatient admission were treated randomly either fully inpatient or in the acute day hospital. As a pilot study, 44 patients were admitted to the study. Evidence of efficacy could be provided for both treatment settings based on significant reduction in psychopathological symptoms and improvement in functional level in the course of treatment. There were no significant differences between the two settings in terms of external assessment of symptoms, subjective symptom burden, functional level, quality of life, treatment satisfaction, and number of treatment days. Treatment in the day hospital was about 45% cheaper compared to inpatient treatment. The results show that acutely ill psychiatric patients of different symptom severity can be treated just as well in an acute day hospital instead of being admitted to the hospital. In addition, when direct treatment costs are considered, there are clear cost advantages for day hospital treatment.

## Introduction

Since the 1970s, psychiatric day hospitals have become increasingly common (1–3). In the meantime, a large number of different psychiatric day hospitals exists. In order to categorize different tasks of day hospital treatment in psychiatric care, a division into four main areas has been proposed: day hospital as an alternative to full inpatient treatment, day hospital as a follow-up treatment after inpatient stay, day hospital as an extension of outpatient treatment, and day hospital as a rehabilitation facility (4).

Day hospital treatment closes the gap between outpatient care and full inpatient hospital admission. Its indication range therefore includes patients whose need for treatment and support is on the one hand to great for a purely outpatient setting and who on the other hand are not necessarily dependent on inpatient treatment (e.g. due to a pronounced deficit in self-care caused by illness or due to acute suicidal tendencies) (2, 5). In direct comparison to full inpatient treatment, advantages can be expected for day hospital treatment on two levels:

Firstly, it can be assumed that the individual patient will benefit from remaining in his or her familiar social environment despite comprehensive therapy. Day hospital treatment facilitates the use and development of the resources available in the social environment (6). In addition, it offers the advantage that existing problems and conflicts in the personal environment can be integrated into the treatment (7). Possible strategies and solutions can subsequently be accompanied therapeutically in small steps. After admission to inpatient treatment, however, our clinical experience has shown that these problems and conflicts tend to become less urgent and important. This subsequently often leads to a lack of treatment or merely the development of a theoretical solution. After discharge from inpatient treatment the patient is then commonly confronted with the problems and conflicts that exist in his personal environment. He may then be unable to implement the solution worked out in the clinical setting, and as a result, the symptoms may quickly exacerbate after discharge from inpatient treatment.

Second, economic advantages are to be expected, as the day hospital requires fewer resources (e.g. no costs for overnight stays, no staff for night duty) for a comparable range of therapies (8, 9). The cost aspect of treatment has become increasingly relevant in recent years owing to the increasingly limited resources in the health care system.

It was not until the 1990s that several randomized controlled trials on the efficacy of day hospital treatments were successfully carried out (6–12). Previously, only naturalistic observations on the effectiveness of day hospital treatment existed (5), and a first randomized study in Great Britain failed to recruit a sufficient number of patients (13). Most studies showed that in both treatment settings—inpatient and day patient—a significant reduction of the psychopathological symptoms existing at admission was achieved, but there was no difference in efficacy between the two forms of treatment (6, 8, 10–12). Furthermore, individual studies have found aspects in which day hospitals performed significantly better than inpatient treatment. The group of Wiersma (6) was able to show that both patients and their relatives showed a greater acceptance of day hospital treatment and that the social reintegration of patients from day hospitals was better. In two studies, the duration of day hospital treatment was significantly shorter (8, 9). One study investigated how strongly patients felt restricted by the treatment and found that day hospital treatment was perceived as less restrictive (7).

A first Cochrane meta-analysis from 2003 comparing day hospital treatment versus inpatient hospital admission for acute psychiatric disorders was based on randomized trials from 1964 to 2000 (14). A total of 1568 patients were randomly treated either in an inpatient setting or in a day hospital. The meta-analysis found no differences between the two forms of treatment with regard to the improvement of the social functional level, the number of treatment days, and the rate of readmission after discharge. However, the patients in day hospital treatment showed a significantly faster decrease in psychopathological symptoms and reported higher treatment satisfaction. In 2011 (15), the Cochrane meta-analysis was revised to include 46 publications based on a total of ten randomized trials with a total of 2685 patients from 1964 to 2007. Four of the included studies were conducted in the USA, three in Great Britain, two in the Netherlands, and one as an EU multicenter study (Germany, Great Britain, Poland, Slovakia, and the Czech Republic). For most of the parameters studied (e.g. reduction of psychopathological symptoms, rate of resumption after discharge, unemployment at the end of the study, and quality of life), no differences were found between the two treatment settings. The advantages of the day hospital in terms of treatment satisfaction and the faster decline of psychopathological symptoms, which were found in the first version of 2003 could not be confirmed in the larger sample. On the contrary, a longer average duration of treatment was actually found for the day hospital. The authors of the Cochrane analysis came to the conclusion that the treatment of acute psychiatric patients in a day hospital is as effective as inpatient treatment.

As already mentioned above, it can generally be expected that the treatment costs of day hospital treatment will be lower than those of full inpatient hospital treatment. The Cochrane meta-analysis, which compares day hospital treatment versus inpatient hospital admission in acute psychiatric conditions (15), takes four studies which also investigated treatment costs into account. Three of these studies were conducted in the United Kingdom (8, 10, 16) and one in the United States (9). The cost advantage for day hospital treatment was between 33.5% and 49.6% when only the index treatment was considered. If the costs for medical treatment and social care outside the index treatment were also included, the cost advantage for the day hospital fell to a range from 20.9% to 36.9%. The results on treatment costs from the EU multicenter study were not included in the Cochrane analysis, as some of these results were only published in German (17). The EU study revealed country-specific differences in treatment costs. The German study center showed a cost advantage of day hospital treatment, whereas the British study center found that day hospital treatment was more expensive than inpatient treatment (17, 18). This comparison is problematic because the authors of the English evaluation included indirect treatment costs such as accommodation costs. These costs accounted for about a quarter of the total costs of day hospital patients. In addition, there was also only a relatively small cost advantage for the direct costs of day hospital treatment, as this had a more intensive therapeutic offer compared to inpatient treatment (18). In contrast, the German evaluation considered only the direct treatment costs. However, not only the costs of the index treatment (day hospital versus full inpatient treatment), but also the three-month period before and after the index treatment were considered (17).

Overall, the studies show a cost advantage for day hospital treatment if only the direct treatment costs are considered. However, as soon as indirect costs are also included, this statement cannot be confirmed at present.

The initial situation for the opening of the Zurich Acute Day Hospital was the expansion of the psychiatric care area of the Psychiatric University Hospital Zurich by two additional city districts with a total of almost 100,000 inhabitants. Instead of the two additional wards required to provide adequate psychiatric care in these two city districts, it was decided to open only one ward and, for the first time in Switzerland, to set up an acute day hospital which is open every day of the week (19). The primary objective of the acute day hospital is thus to treat patients who would otherwise have to be admitted as inpatients. On the one hand, this should relieve the burden on the inpatient wards, and on the other hand, patients can benefit from the advantages of day hospital treatment.

This evaluation study investigates the question of whether certain acutely ill psychiatric patients who are in need of inpatient treatment can be adequately treated in the acute day hospital instead of inpatient treatment. In addition, it is to be examined whether the postulated cost advantages for inpatient treatment are also available in the Swiss health system. So far, there has been no randomized comparison of inpatient and day patient treatment for acute psychiatric disorders in the Swiss health care system. The present study therefore examines for the first time whether the findings gained in other countries concerning the efficacy and efficiency of psychiatric day hospital treatment can be transferred to Switzerland.

On the basis of the results published so far on the efficacy and efficiency of psychiatric day hospitals, the following hypotheses were formulated:

1. Psychopathology: at the time of discharge, there is no difference in the improvement of mental symptoms between the inpatient and day-care treatment groups.
2. Treatment satisfaction: the patients treated in the acute day hospital are more satisfied compared to those treated in hospital.
3. Length of hospitalization: the inpatient and day patient treatment groups do not differ in the number of treatment days.
4. Treatment costs: the costs of treatment in the group treated in the acute day hospital are significantly lower compared to the group treated in the hospital.

## Methodology

During the study period from October 2011 to March 2013, all patients who came to the hospital with a referral for an inpatient admission were to be tested for the prerequisites for inclusion in the study. If the inclusion criteria were fulfilled, oral and written information about the study was provided. After a patient had agreed to participate in the study, including randomization, the patient was assigned to one of the two treatment groups by lot. Lots were pseudo-randomized by a neutral person who was not involved in the study by always bundling 10 lots (5 × inpatient and 5 × day hospital). First ten lots were put into the lot pot, as soon as only three lots were left in the pot, a new bundle of 10 lots was added. This procedure was chosen to achieve an equal distribution of patients between the two treatment settings. Since the study was limited in time and not by a maximum number of patients included, there would otherwise have been a risk of having a significantly different number of patients in the two groups. The study was approved by the cantonal ethics commission (EK: 2011-0015/3).

Integrated psychiatric treatment including milieu therapeutic elements as well as pharmacotherapy and psychotherapy were provided both in the acute day hospital and in the inpatient ward. In addition to medical visits, the range of therapies offered consists mainly of group therapies and therapeutic one-on-one consultation, which are provided by the multidisciplinary staff. The therapeutic teams included doctors, psychologists, and qualified specialists from the fields of nursing, social work, occupational therapy, as well as movement and expression therapy. The therapeutic group programs included occupational therapy, cognitive training, expressive and creative therapy, movement and relaxation therapy, psychoeducation, psychotherapeutic groups, sports groups, and milieu-therapeutic offers, such as outdoor activities and a cooking group. The focus of the psychotherapeutic groups was stress management, problem-solving skills, as well as increasing social and emotional competence.

The data were recorded at the beginning of the treatment and at the time of discharge. Within 24 h after admission, the psychopathological symptoms and the general functional level were recorded using standardized recording instruments. If possible, this was done directly by the receiving physician during the initial examination. The standardized external assessment of the severity of the symptoms was supplemented by a self-assessment of the state of health. During the first week of treatment, the study participants received questionnaires to record subjective symptom burden, quality of life, and recovery, i.e., individual recovery potential. At the time of discharge, a standardized assessment of the current psychopathological findings was carried out by a study staff member who was not involved in the treatment and patients received questionnaires on subjective symptom burden, quality of life, treatment satisfaction, and recovery potential.

### Inclusion and Exclusion Criteria

The study included female and male patients with referral to an inpatient admission at the Psychiatric University Hospital of Zurich for treatment. In addition, the following inclusion criteria had to be met:

Admission to the hospital on a voluntary basis.
Age: 18 to 65 years old
The treatment is based on a psychiatric diagnosis from Chapter V of the ICD-10 F2* to F6* (schizophrenia, schizotypal and delusional disorders, affective disorders, neurotic, stress and somatoform disorders, behavioral disorders with physical disorders and factors, personality and behavioral disorders).
Sufficient language skills (German or Swiss German) of the participating patients.
A written declaration of consent to participate in the study is available.

Exclusion criteria for participation in the study were:

The presence of an organic mental disorder (ICD-10: F0*) or an addictive disorder (ICD-10: F1*).
An existing pregnancy.
Acute self or external hazard without the ability to form an alliance that makes day hospital treatment impossible.
The initiation of treatment on the basis of a coercive measure (“*Fürsorgerische Unterbringung*”).
Comorbid somatic disease requiring long-term care that makes day hospital treatment impossible.

### Recruitment of the Study Sample

**Figure 1:**
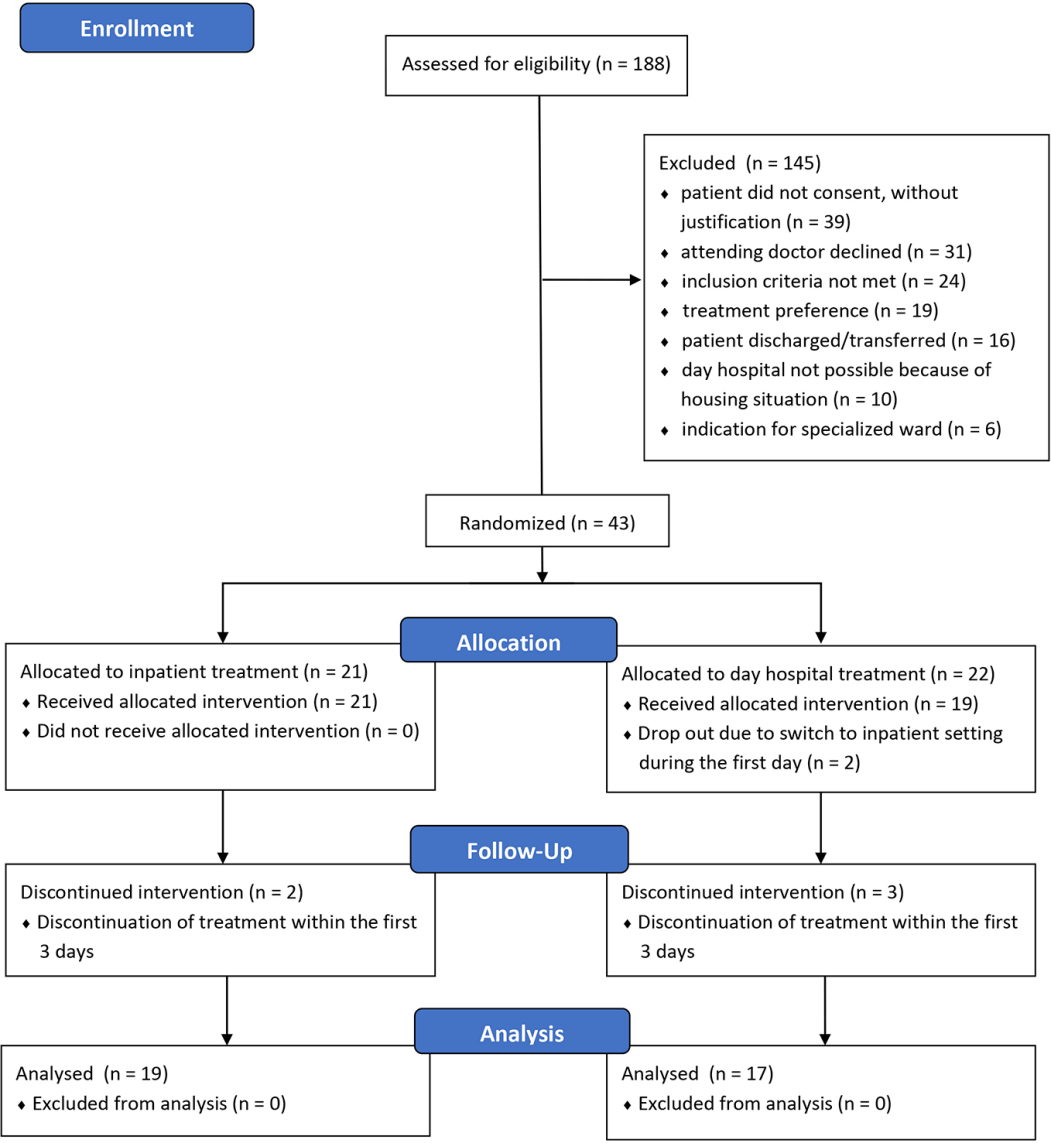
Recruitment of the study sample and randomization according to the CONSORT statement (www.consort-statement.org).

A total of 188 female and male patients were checked for the possibility of inclusion in the study (**Figure 1**). 144 of these patients (76.6%) were not included: 31 patients were not included in the study, as the directly treating physician or senior physician did not consider inclusion in the study to be appropriate as inpatient treatment seemed necessary. Further 39 patients could not be included because they did not give their consent, without justification. In 24 patients, the inclusion criteria were not met, and 19 patients refused randomization because they wanted to choose one of the two treatment options (mostly day hospital). 16 patients were discharged or transferred to another hospital prior to inclusion. Ten patients could not be included due to their housing situation (no fixed residence or too far away from their residence) and six patients were admitted directly to a specialized ward (e.g., mother-child />ward or ward for early detection and treatment of psychoses).

Of the 44 patients enrolled in the study, only 43 were randomized because one patient had withdrawn his previous consent. Randomization assigned 21 patients to full inpatient treatment and 22 to day hospital treatment. Thirty-six patients participated in the study until discharge. Hence, the data of 19 patients from full inpatient treatment and 17 patients from day-care treatment could be included in the evaluation.

### Data Collection

The severity of the clinical symptoms and the functional level were assessed by trained investigators. Psychopathological symptoms—Brief Psychiatric Rating Scale (BPRS) (20)—the symptom burden and social functional level—Health of the Nation Outcome Scales (HoNOS) (21, 22)—the global severity of the disease—Clinical Global Impression Severity Score (CGI-S) (23)—and the global level of functioning—Global Assessment of Functioning (GAF) (24)—of the participating patients were recorded both at admission and discharge. In addition, quality of life, satisfaction, subjective symptom burden, and recovery style were assessed using self-assessment tools (**Table 1**). Subjective symptom burden was assessed using the Symptom Check List (SCL-10) (27) and the Outcome Questionnaire (OQ-45) (28). The OQ-45 also assessed the subjectively experienced difficulties in interpersonal relationships and social integration. Quality of life was measured with the Manchester Short Assessment of Quality of Life (MANSA) (25) scale. The five factors of the Recovery Assessment Scale (RAS-24) (29): personal confidence and hope, willingness to ask for help, goal and success orientation, reliance on others, and no domination by symptoms were used to measure recovery style. At the time of discharge, patient satisfaction was measured once with the Client Satisfaction Questionnaire (CSQ-8) (26).

**Table 1:** Overview of the rating instruments and time of acquisition.

Rating instruments	Admission	Discharge
Brief Psychiatric Rating Scale (BPRS) (20)	X	X
Health of the Nation Outcome Scales (HoNOS) (21, 22)	X	X
Clinical Global Impression Severity Score (CGI-S) (23)	X	X
Global Assessment of Functioning (GAF) (24)	X	X
**Self-rating instruments**		
Manchester Short Assessment of Quality of Life (MANSA) (25)	X	X
Client Satisfaction Questionnaire (CSQ-8) (26)		X
Symptom Check List (SLC-10) (27)	X	X
Outcome Questionnaire (OQ-45) (28)	X	X
Recovery Assessment Scale (RAS-24) (29)	X	X

### Calculation of Direct Treatment Costs

The direct treatment costs were recorded on the basis of data from the Department of Finance and Business Administration at the Psychiatric University Hospital Zurich. The available data from the contribution margin calculation did not allow the real costs incurred to be recorded for each individual case of treatment. Thus, it is not recorded in detail how much time an employee spends for a particular patient, which makes it impossible to determine the exact personnel costs for the individual case. Material costs, such as medication costs, are only recorded for each ward or day hospital, but not for the individual treatment case. However, it was possible to determine the average costs for a treatment day from the data from cost center accounting and the treatment days performed. The evaluation study was carried out from October 2011 to May 2013, so the year 2012 was used as basis for the cost calculation. For this purpose, the acute day hospital cost center and the cost center for an open general psychiatric ward were evaluated. The costs for the complete calendar year 2012 were taken as the basis and then divided by the number of treatment days performed in the respective unit (day hospital, open ward) during this year. Only the treatment days were taken into account for this calculation. For the day hospital, this means that the days between admission and discharge were not taken as the basis for determining the treatment days, but only the days on which the patient took advantage of the day hospital offer. In cost accounting, both direct operating expenses and personnel costs, material costs (e.g., drugs, material for therapies) and services provided by third parties (e.g., somatic specialist departments) as well as allocations (e.g., pro rata administrative costs, cleaning, building maintenance) were taken into account. The calculation revealed that total costs per treatment day were 1.73 times higher for open ward compared to day hospital.

### Data Processing and Statistics

Most of the data was initially collected on paper—either by the participating patients who completed the self-rating questionnaires or by the investigators who recorded the responses to the structured interviews and clinical observations in the third-party rating forms. For further analysis, the data were transferred to a SPSS database (Statistical Package for the Social Sciences, IBM, Version 24). The treatment duration information was taken from the computerized hospital information system and also transferred to the SPSS database. To calculate the direct treatment costs, the number of treatment days was multiplied by the daily rate for the respective treatment.

The statistical analysis was also performed using SPSS (IBM, Version 24) with a significance level of α = 0.05. Group comparisons (day hospital versus inpatient) as well as longitudinal comparisons (admission time versus discharge time) were calculated. The gender distribution (male, female), the characteristic occupational activity of at least 40% and the frequency of diagnoses were examined as categorical data using the chi-square test. Using the Kolmogorov-Smirnov test, the normal distribution of most data was confirmed, so that the T-test could be used for simple group comparisons except for the parameters schooling in years and treatment cost (Mann-Whitney *U* test). To record the changes in the course of treatment, variance analyses (ANOVA) were calculated using the internal subject factor time (admission, discharge) and the intermediate subject factor treatment setting (day hospital, inpatient).

## Results

### Study Sample

The data of 36 patients with completed treatment in a randomly assigned setting (19 fully inpatient and 17 day hospital) could be included in the evaluation. There were no significant differences between the two treatment groups in terms of age, gender distribution, school education in years and occupational activity (**Table 2**).

**Table 2:** Age and school education in years, mean *(± standard deviation)*, p-values (t-tests, inpatient versus day hospital); gender (male/female), and occupational activity, p-values (Chi-square-tests, inpatient versus day hospital).

	Inpatient	Day hospital	p-value
Age (years)	38.2 (± *12.3*)	41.2 (± *11.6*)	0.45 (n.s.)
Gender (m:f)	11:8	8:9	0.74 (n.s.)
School education (years)	11.5 (± *3.0*)	10.8 (± *2.7*)	0.71 (n.s.)
Occupational activity (at least 40%)	7 of 19	8 of 17	0.54 (n.s.)

n.s., not significant.

The psychiatric treatment diagnoses of the patients included were distributed among four diagnostic groups of Chapter V (mental and behavioral disorders) of the International Classification of Diseases (ICD-10) (30). The Chi-square test showed that there were no significant differences in the frequency distribution of diagnoses between the two treatment settings (**Table 3**).

**Table 3:** Distribution of the psychiatric treatment diagnoses, itemized according to the diagnostic groups of the International Classification of Diseases (ICD-10), global p-value (Chi-square-test, inpatient versus day hospital).

Diagnostic group	Inpatient	Day hospital	p-value
F2: schizophrenia, schizotypal, and delusional disorders	4	7	0.21 (n.s.)
F3: mood [affective] disorders	10	6	
F4: Neurotic, stress-related, and somatoform disorders	4	1	
F6: Disorders of adult personality and behavior	1	3	

n.s., not significant.

### Psychopathology and Functional Level

The mean values of psychopathological symptoms are shown in **Table 4**. At the time of admission, there were no significant group differences with regard to the existing psychopathological symptoms and symptom burden. On average, the patients randomly assigned to the day hospital had a somewhat higher global level of functioning, but the difference did not reach the statistical significance level of p < 0.05.

**Table 4:** Brief Psychiatric Rating Scale (BPRS) and Health of the Nation Outcome Scales (HoNOS), mean sum scores *(± standard deviation)*; Clinical Global Impression Severity Score (CGI-S) and Global Assessment of Functioning (GAF), means; p-values (t-tests, inpatient versus day hospital).

	Admission			Discharge		
	Inpatient	Day hospital	p-value	Inpatient	Day hospital	p-value
BPRS	16.9 (± *8.0*)	16.7 (± *9.6*)	0.95 (n.s.)	12.9 (± *10.3*)	12.5 (± *11.1*)	0.89 (n.s.)
HoNOS	15.6 (± *5.9*)	16.5 (± *7.3*)	0.71 (n.s.)	11.6 (± *6.8*)	9.6 (± *7.9*)	0.41 (n.s.)
CGI-S	4.8 (± *0.6*)	4.4 (± *0.6*)	0.10 (n.s.)	3.3 (± *0.8*)	3.5 (± *0.64*)	0.64 (n.s.)
GAF	40.4 (± *19.6*)	49.7 (8.7)	**0.08** (trend)	63.9 (± *16.8*)	63.0 (± *15.2*)	0.86 (n.s.)

n.s., not significant.

At the time of discharge, there were also no significant differences between the two treatment settings with regard to psychopathological symptoms and the functional level. The analysis of variance (ANOVA) with the inner subject factor *time* and the intermediate subject factor *treatment setting* showed a significant effect on the factor *time* for all four scales: BPRS (F = 9.66; p = 0.004), Ho-NOS (F = 37.46; p < 0.0001), CGI (F = 39.68; p < 0.0001), and GAF (F = 34.94; p < 0.0001). This means that both treatment settings were effective and led to a significant decrease in symptom burden at the time of admission. In addition, the functional level could be significantly increased in both treatment settings.

**Figure 2:**
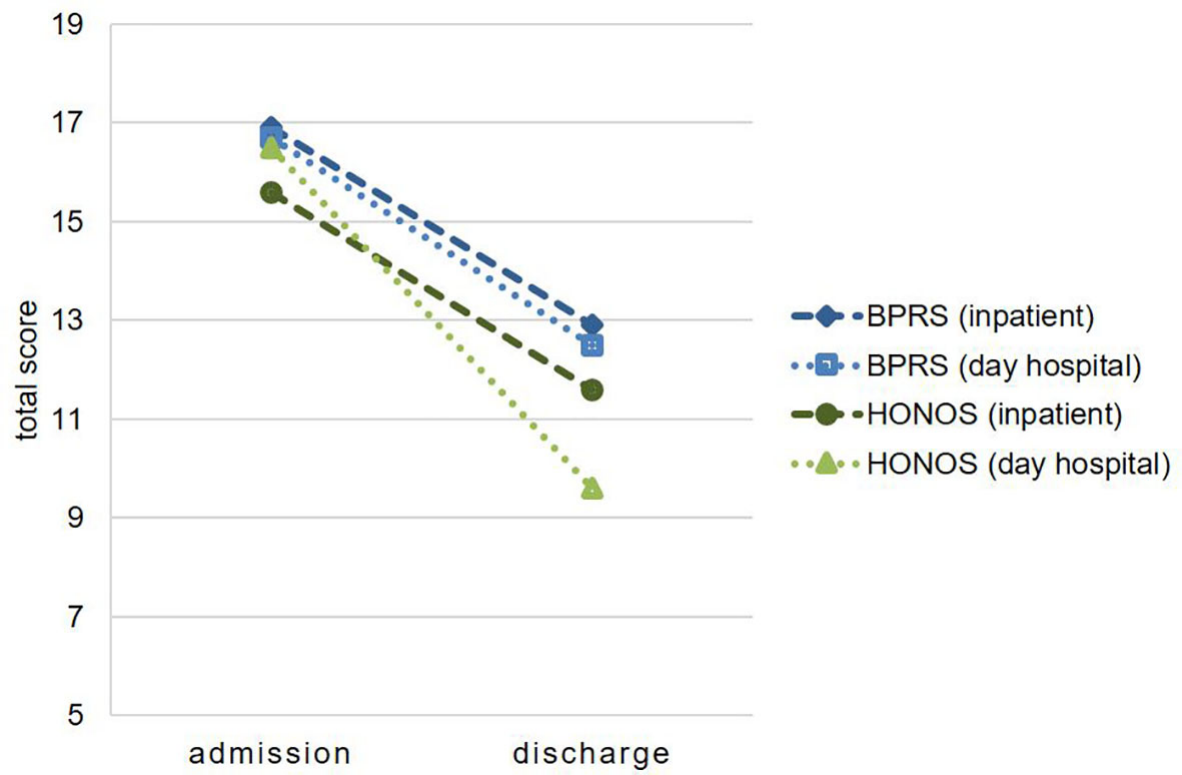
Change in psychopathology under treatment. BPRS, Brief Psychiatric Rating Scale; HoNOS, Health of the Nation Outcome Scales (means of total scores).

The factor *treatment setting* alone was not significant for any of the four scales, but a trend was found for the interaction *time* x *treatment setting* for the HoNOS (F = 3.58; p = 0.067). The HoNOS thus indicates a relatively stronger decrease in symptom burden in the day hospital treatment group (**Figure 2**). However, the three other scales support the statement that both treatment settings were equally effective with regard to the improvement of psychopathological symptoms and functional level.

### Subjective Symptom Burden and Social Interaction

There were no significant differences in the subjectively experienced symptom burden measured with the SCL-10 between the two treatment groups at the beginning of treatment or at the time of discharge (**Table 5**). The analysis of variance (ANOVA) with the inner subject factor *time* and the intermediate subject factor *treatment setting* showed a significant reduction of the SCL-10 sum score during the course of treatment (F = 5.19; p = 0.030), whereby there were no significant differences between the two forms of treatment.

**Table 5:** Symptom Check List (SCL-10) and Outcome Questionnaire (OQ-45), mean sum scores *(± standard deviation*), p-values (*t*-tests, inpatient versus day hospital).

	Admission			Discharge		
	Inpatient	Day hospital	p-value	Inpatient	Day hospital	p-value
**SCL-10**	16.8 (± *9.9*)	19.3 (± *8.2*)	0.44 (n.s.)	12.4 (± *8.6*)	14.3 (± *14.7*)	0.64 (n.s.)
**OQ-45**						
Symptom burden	45.5 (± *19.6*)	42.1 (± *14.2*)	0.68 (n.s.)	43.7 (± 41.4)	40.4 (± 23.9)	0.81 (n.s.)
Relationship	17.9 (± *7.4*)	16.0 (± *7.0*)	0.58 (n.s.)	19.5 (± 15.0)	14.7 (± 9.1)	0.88 (n.s.)
Social integration	14.1 (± *5.8*)	12.3 (± *7.0*)	0.50 (n.s.)	11,7 (± 7.1)	9.6 (± 4.4)	0.38 (n.s.)
Total score	75.7 (± *30,0*)	69.0 (± *27,1*)	0.66 (n.s.)	70,7 (± 33.9)	57.3 (± 31.4)	0.41 (n.s.)

n.s., not significant.

The evaluation of the OQ-45 also showed no significant differences between the two treatment settings (**Table 5**).

The analysis of variance (ANOVA) with the inner subject factor *time* and the intermediate subject factor *treatment setting* showed a significant result for the factor time (F = 5.16; p = 0.036) only for the sub score social integration. In other words, the subjectively experienced problems in social integration at the time of admission decreased during the course of treatment without any difference between the two treatment settings (setting, F = 1.03; p = 0.325).

### Recovery

There was no significant difference in terms of the recovery style between the two treatment settings at the time of admission or discharge. However, the target and success orientation at the time of admission was less pronounced among patients in the day hospital at the trend level compared to fully inpatient treatment.

An analysis of variance (ANOVA) with the internal subject factor *time* and the intermediate subject factor *treatment setting* showed a significant result only for the scale goal and success orientation, at least at the trend level. The trend showed a difference between the two treatment groups (setting: F = 3.41; p = 0.075). The time factor (F = 2.96; p = 0.096) was significant at the trend level, indicating a decline in goal and success orientation in the course of treatment (**Table 6**).

**Table 6:** Recovery Assessment Scale (RAS-24), mean scores *(± standard deviation*, p-values (t-tests, inpatient versus day hospital).

RAS-24	Admission			Discharge		
	Inpatient	Day hospital	p-value	Inpatient	Day hospital	p-value
Personal confidence and hope	3.65 (± *0.93*)	3.52 (± *0.76*)	0.67 (n.s.)	3.60 (± *1.07*)	3.47 (± *0.85*)	0.68 (n.s.)
Willingness to ask for help	4.06 (± *1.17*)	3.85 (± *0.83*)	0.58 (n.s.)	4.25 (± *0.85*)	3.88 (± *0.98*)	0.24 (n.s.)
Goal and success orientation	4.81 (± *2.71*)	3.72 (± *0.75*)	**0.08** (trend)	3.96 (± *1.06*)	3.56 (± *0.59*)	0.18 (n.s.)
Reliance on others	3.85 (± *0.86*)	4.21 (0.59)	0.17 (n.s.)	3.91 (± *0.67*)	3.97 (± *0.80*)	0.83 (n.s.)
No domination by symptoms	3.06 (± *1.07*)	3.33 (± *0.54*)	0.40 (n.s.)	3.27 (± *0.86*)	3.19 (± *0.79*)	0.77 (n.s.)

n.s., not significant. bold = significant p < 0.05.

### Quality of Life and Treatment Satisfaction

Descriptively, the MANSA showed a slightly higher subjective quality of life in the group of patients treated in the day hospital at the time of admission and discharge (see **Table 7**). In addition, the descriptive data point to an improvement in subjective quality of life during treatment in both settings. However, an analysis of variance (ANOVA) with the inner subject factor *time* and the intermediate subject factor *treatment setting* showed that this change was not significant (F = 1.91; p = 0.178).

**Table 7:** Manchester Short Assessment of Quality of Life (MANSA), mean scores *(± standard deviation*, and Client Satisfaction Questionnaire (CSQ-8), mean sum scores (± standard deviation), p-values (t-tests, inpatient versus day hospital).

	Admission			Discharge		
	Inpatient	Day hospital	p-value	Inpatient	Day hospital	p-value
**MANSA**	3.95 (± *0.99*)	4.27 (± *1.09*)	0.40 (n.s.)	4.36 (± *1.30*)	4.55 (± *0.69*)	0.69 (n.s.)
**CSQ-8**				25.24 (± 5.92)	28.56 (± 5.81)	0.11 (n.s.)

n.s., not significant.

On average, the patients were satisfied to very satisfied with the treatment measured with the CSQ-8. Although the descriptive values point to an advantage for day hospital treatment, the difference between the two treatment settings was not significant (**Table 7**).

### Treatment Duration and Direct Treatment Costs

The total duration of treatment, from the date of admission to discharge, varied widely from 4 days to 78 days for inpatient treatment and from 16 days to 119 days for day hospital treatment. However, the actual treatment days are relevant for the calculation of treatment costs, as only these days generate costs and are billed to the health insurances. While the total duration of treatment in the day hospital was significantly longer than in the fully inpatient treatment, the number of treatment days did not differ significantly (**Table 8**).

**Table 8:** Total duration of treatment: days between admission and discharge, treatment days, and total treatment costs (in CHF), mean scores *(± standard deviation*, *p*-values (t tests, inpatient versus day hospital).

	Inpatient	Day hospital	p-value
Total duration of treatment (in days)	31.5 (± *21.3*)	55.7 (± *32.2*)	**0.011**
Treatment days	31.5 (± *21.3*)	29.5 (± 15.3)	0.75 (n.s.)
Total treatment costs (in CHF)	24549.47 (± *16644*)	13291.24 (± *6915*)	**0.012**

n.s., not significant. bold = significant p < 0.05.

**Figure 3:**
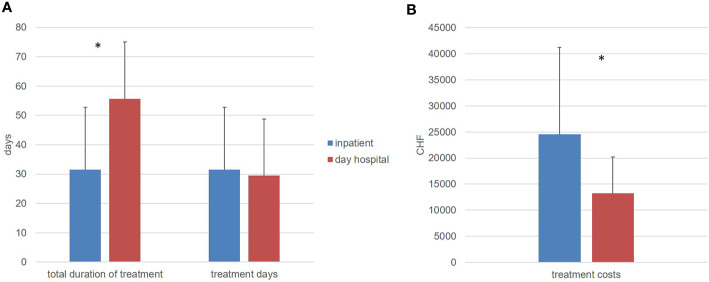
**(A)** total duration of treatment in days and treatment days (means and standard deviation). **(B)** direct treatment costs in swiss franc (CHF) (means and standard deviation), *significant difference (p < 0.05).

To calculate the treatment costs, the treatment days were multiplied by the costs per treatment day. In the fully inpatient setting, the costs in Swiss francs (CHF) for a single case ranged between CHF 3120 and CHF 60840 and in the day hospital between CHF 3608 and CHF 24354. The Mann-Whitney-U-test (U = 34.000; p < 0.0001) showed a significant cost difference between the two treatment settings for the benefit of day hospital treatment (**Figure 3**).

## Discussion

The main aim of this study was to find answers to the following two questions: First, whether acutely ill psychiatric patients with existing indication for inpatient treatment can be treated just as effectively in the Zurich Acute Day Hospital and second, whether inpatient treatment causes lower treatment costs in the Swiss health care system than inpatient treatment. Under the assumption that both forms of treatment—inpatient and day-care—are equally suitable for treating acutely mentally ill patients, we hypothesized that, at the time of discharge, no differences concerning the improvement of psychological symptoms between the inpatient and day-care treatment groups can be found. This hypothesis is confirmed by the results of the study.

Statistical testing showed that patients in the two treatment groups did not differ in age, gender, education, current level of employment, and distribution of primary diagnosis, despite randomized allocation by lot. Also, with regard to psychopathology, the two treatment groups did not differ in terms of symptom severity at the time of inclusion into the study.

Thus, the symptom severity measured in the rating instruments collected by the trained investigators was comparable in both treatment groups, and even the subjective symptom burden assessed by the patients themselves did not differ between the two settings. In the global level of functioning (GAF), there was a slightly higher functional level in the inpatient treatment group at the time of admission, but this difference was not significant.

After it was shown that both groups were comparable at the start of treatment, the next step was to examine whether significant differences could be demonstrated after completion of treatment compared to the start of treatment on the one hand and between the two treatment settings on the other. Even at the time of discharge from the respective treatment, no group differences in symptom severity and functional level could be found between the two settings. Fortunately, in both treatment settings there was a significant decrease in psychopathological symptoms and an improvement in global level of functioning during the course of treatment. With regard to the decrease in psychopathological symptoms due to treatment, only the HoNOS showed a difference between the two treatment settings at trend level with a relatively stronger decrease in symptom burden in the day hospital treatment group. However, this effect did not reach the significance level. Thus, in the present study, both forms of treatment showed an equally good efficacy with regard to symptom reduction.

The present result that both treatment settings are almost equally effective in suitable patients—i.e., in the absence of contraindications for day patient treatment—is congruent with the randomized studies published so far from other countries (6, 8, 10–12, 31, 32).

The survey of the individual recovery potential with the Recovery Assessment Scale (29) revealed an unexpected result. In both treatment settings, patients received both individual and group psychotherapy, which also includes the promotion of general resilience and resources for coping with the disease. Under the assumption that this should have an impact on the recovery potential, an increase in the RAS-24 values would have been expected. However, four of the factors of the RAS-24 showed no significant difference. It is possible that changes in the recovery potential can only be assessed over a longer period of time, an older study on the long version of RAS showed a high retest reliability over a period of 14 days (33). Nevertheless, the present study showed a decline in the factor goal and success orientation during the course of treatment on trend level. One explanation for this unexpected decline in goal orientation could lie in the content of psychotherapy. Techniques for coping with stress were also taught in both treatment settings. This includes exercises to reduce inner demands on oneself in favor of strengthening regenerative activities (34). A successful reduction of the demands on oneself could possibly lead to a reduction of the goal and success orientation. Hence, the observed decline in the factor “goal and success orientation” also includes a positive aspect in the sense of stress reduction.

As a second hypothesis, based on the study by Dick et al. (16), it was assumed that the patients treated in the acute day hospital were more satisfied with the treatment than those treated fully in an inpatient setting. This hypothesis could not be confirmed: Although the descriptive evaluation of the CSQ-8 indicated a greater treatment satisfaction among the patients of the acute day hospital, this difference was not significant. Also, with regard to the subjective quality of life recorded with the MANSA, only a descriptive positive effect of both treatment settings was observed, which, however, was also not statistically significant. It is possible that the effect strength is rather low in both cases and could therefore only reach a significant level in a considerably larger patient group.

With regard to the duration of treatment, there was a broad spread of 4 to 119 days between admission and discharge across the entire patient population. This wide spread is not surprising based on clinical experience. Even with the same treatment diagnoses and similar symptoms, the duration of treatment often differs significantly between individual patients (35). For example, it has not yet been possible to establish flat-rate hospital fees for psychiatric diagnoses based on average treatment expenditure, analogous to the somatic DRG system (Diagnosis Related Groups).

The comparison of the two treatment groups indicated that the total duration of treatment—the period from the day of admission to the day of discharge—was significantly longer in the day hospital group. This difference reached a significant level and is consistent with previously published studies, which also showed a significant longer total treatment duration for day hospital compared to full inpatient treatment (15). In the present study, the focus was deliberately placed on treatment days, since only these are charged to health insurances, and no costs are incurred for days without treatment. With regard to the duration of index treatment, the hypothesis was made that inpatient and day patient treatments do not differ in terms of the number of treatment days.

The treatment duration for the present study was almost identical in both settings regarding the number of real treatment days. This confirmed the hypothesis regarding the number of treatment days. In a descriptive comparison, the average number of days of inpatient treatment was even lower, but this difference was not significant due to the high variance. This result is not congruent with the meta-analysis by Marshall et al. (15), which also found an advantage, albeit very small, for inpatient treatment for the number of treatment days. Only the study conducted in the USA by Sledge et al. (9, 11) also found a shorter treatment duration for day hospital treatment.

The fourth hypothesis of the present study related to treatment costs, assumed a significant cost advantage for treatment in the acute day hospital. This hypothesis was also confirmed. Despite the wide spread of treatment costs per treatment case, a statistically significant cost advantage for treatment in the acute day hospital could be demonstrated.

### Strengths and Limitations

The present study is based only on the direct costs of index treatment. If indirect treatment costs are also considered, it can be assumed that this cost advantage will decrease. The meta-analysis by Marshall et al. (15) assume the reduction of the cost advantage of day hospital treatment at 25% to 37%. The study by Priebe et al. (18) even showed a cost disadvantage for day hospital treatment if indirect costs such as accommodation were included. In our opinion, however, a valid calculation of indirect medical costs is hardly possible. Tools developed specifically for recording the use of social services, such as the Client Sociodemographic and Service Receipt Inventory (36), are also simply based on the patient's reports, e.g. “How many appointments with the psychiatrist did you have in the last 12 months?” “Average duration per appointment,” or “How many contacts with the police took place?” From this information, accurate costs should then be calculated for the use of services.

It is understandable that day hospital treatment incurs costs that are not incurred in a fully inpatient setting, such as the costs for daily journeys between home and day hospital. However, the share of travel costs in indirect medical costs is to be regarded as relatively low. Much more serious are sickness-related incapacity to work which represents a significant proportion of indirect medical costs (37, 38). Unfortunately, the present study did not record when patients returned to work and how many days of sick leave they had in total. It is striking that, despite the age range from 18 years to 65 years, only 42% of the study's participants had a regular occupational activity of at least 40% prior to the outbreak of the acute symptoms leading to hospital admission.

The significance of the present study is unfortunately limited by the small number of patients included. The low number of cases is further reduced because only the data of the completers were recorded, and thus an Itent-To-Treat analysis was not possible. Even though 188 patients were examined for the possibility of a study inclusion in the course of the recruitment phase, only 43 patients could be randomly included. In addition to the non-fulfillment of the inclusion criteria and the lack of consent to the study inclusion, 31 patients in this study also showed the phenomenon that the directly treating physicians rejected the study inclusion for their patients because they did not consider the two forms of treatment to be equally suitable, as already described by Platt et al. (6) as one of the main reasons for the failure of their study. In addition, every tenth patient suitable for participating in this study refused to be included because they preferred one of the two forms of treatment and did not want to leave this to chance. It is possible that the expectations of the patients also played a role here, so that the patients had either come directly to the hospital as an emergency or had been assigned by the general practitioner to inpatient treatment. Consent to the study thus also meant, depending on the lot decision, not to receive the expected inpatient treatment, but rather a previously little-known day-care treatment. Therefore, a potential selection bias cannot be excluded in the present study. Another limitation is that the study was carried out already in the year 2012. It can therefore be assumed that treatment costs in both settings have increased in the meantime.

Despite the low number of participants, a significant treatment effect could be proven in both therapy settings, which speaks for a high effect strength of psychiatric acute treatment. The presumed cost advantage for day hospital treatment also reached a significant level despite of the small sample. In our opinion, however, the present study should be repeated with a larger sample in order to provide more evidence for the cost-efficacy and to identify minor differences between the two forms of treatment.

## Conclusion

This study is the first to examine the efficacy and efficiency of a psychiatric day hospital in acute care in the Swiss health care system. In summary, the two main questions of the study were answered positively. The results show that patients with acute psychiatric illness for whom there is an indication for inpatient treatment can be adequately treated in an acute day hospital instead of being admitted to the hospital. For both treatment settings a significant proof of efficacy could be proven with regard to the psychopathological symptoms and the individual functional level. Concerning direct treatment costs, psychiatric day hospital treatment offers clear cost advantages compared to inpatient treatment in the Swiss health care system.

## Data Availability Statement

The datasets generated for this study will not be made publicly available. Psychopathological data will be available by request to the corresponding author. Unfortunately, for competitive reasons, we do not have permission to provide detailed cost data.

## Ethics Statement

The studies involving human participants were reviewed and approved by Cantonal Ethics Commission Zurich. The patients/participants provided their written informed consent to participate in this study.

## Author Contributions

KH, WR, and WK designed the study and wrote the protocol. SA, BH, CO, and MK collected the data. KH drafted the manuscript and conducted all analyses. KH, SA, BH, CO, MK, ES, WR, and WK discussed the results, reviewed the manuscript, and made critical revisions. All authors contributed to and have approved the final manuscript.

## Funding

The study was funded by a private Foundation. The funding is in accordance with the legal requirements and was approved by the Ministry of Health of the Canton of Zurich (Switzerland). The funding body did not play any role in study design; in the collection, analysis, and interpretation of data; in the writing of the manuscript; and in the decision to submit the manuscript for publication.

## Conflict of Interest

The authors declare that the research was conducted in the absence of any commercial or financial relationships that could be construed as a potential conflict of interest.
